# A Rare Case of Insulinoma in a Thin, Lean Adult Male: A Case Report

**DOI:** 10.7759/cureus.23414

**Published:** 2022-03-23

**Authors:** Ahmad R. Khan, MD, Muhammad Hayyan Wazir, Salma Waqar, Rizwan Ullah, Ayesha Gul

**Affiliations:** 1 Internal Medicine, Hayatabad Medical Complex Peshawar, Peshawar, PAK; 2 Obstetrics and Gynecology, Mardan Medical Complex, Mardan, PAK

**Keywords:** persistent hyperinsulinemic hypoglycemia, pancreatic disease, pancreatic malignancy, c-peptide, pancreatic insulinoma

## Abstract

Insulinoma is an insulin-secreting tumor that causes hypoglycemia due to inappropriately high insulin secretion. The Whipple's triad, which comprises indications of hypoglycemia (tremor, sweating, irritability, uneasiness, and weakness), plasma glucose concentration <55 mg/dL (3.0 mmol/L), and resolution of symptoms after administration of glucose, is utilized for the determination of insulinoma. In this report, we present the case of a thin, lean, adult male with a BMI of 22, who presented with repetitive episodes of tremor, sweating, weariness, and perplexity that occurred amid fasting and settled with meals, fulfilling Whipple's triad criteria for the determination of insulinoma. The episodes frequently led to seizures. Supervised fasting was carried out, which revealed raised C-peptide levels, low blood glucose, and negative sulfonylurea screen. A computed tomography (CT) scan localized the tumor, and surgical resection was planned.

## Introduction

Insulinoma is a small insulin-secreting tumor that causes hypoglycemia due to inappropriately high insulin secretion. It is a type of functional neuroendocrine tumor (NET) and is exceptionally uncommon with a yearly frequency of 1 to 4 per million per year [1,2]. We present the case of a 20-year-old lean boy with repeated episodes of weakness, fatigue, sweating, and tremors that settled with meals. The gold standard for biochemical diagnosis is an estimation of plasma glucose, insulin, C-peptide, and proinsulin during a 72-hour fast. Up to 99% of insulinomas can be differentiated with the mentioned fasting test [3].

The therapy of choice is surgical resection. Imaging studies, including a CT scan, localize the tumor before resection. The enucleation of insulinoma and partial distal pancreatectomy are the two most widely employed methods [1]. Other choices incorporate laparoscopy, which has comparable results [4]. Both short- and long-term comparable results have been reported in a small number of cases for mechanical enucleation of insulinoma as compared to the laparoscopic approach [5,6].

## Case presentation

For the past three years, a 20-year-old slender, lean adult male complained of increased hunger, tremors, perspiration, palpitation, confusion, and frequent seizures. Within the last four months, these symptoms got worse. According to the attendant, incidents usually happened late at night or in the early hours of the morning while he was fasting, and they lasted anywhere from a few minutes to several hours. The patient denied having any visual field issues or headaches and said he had never abused drugs. His past medical and surgical records were unremarkable.

A young patient lying quietly in bed without distress on general physical examination was found to have scanning speech and horizontal nystagmus, with the rest of the systemic examination results being normal.

High level of blood insulin (185 pmol/L; reference range: 14.01 to 179 pmol/L) and low level of blood glucose (50 mg/dL; reference range: 70-100 mg/dL) were revealed by initial laboratory tests. Other important profiles such as renal, thyroid, hepatic, coagulation, bone, prolactin, and parathyroid were all in the normal range, along with complete blood count. The patient was hospitalized for additional testing due to recurrent hypoglycemia symptoms and a potential pancreatic insulinoma. During a hypoglycemic episode (palpitations, diaphoresis, tremulousness, slurred speech, disorientation, sleepiness) that occurred during prolonged (48-hour) fasting, his insulin and C-peptide levels were examined. Laboratory findings during the event revealed low blood glucose (37 mg/dL), increased blood insulin (190 pmol/L), and high C-peptide (4.66 ng/mL; reference range 1.10 to 4.40 ng/mL) values. Sulfonylurea or meglitinide toxicity was not seen in the blood. Dextrose injection significantly alleviated hypoglycemia symptoms, and Whipple's triad for insulinoma was satisfied.

**Figure 1:**
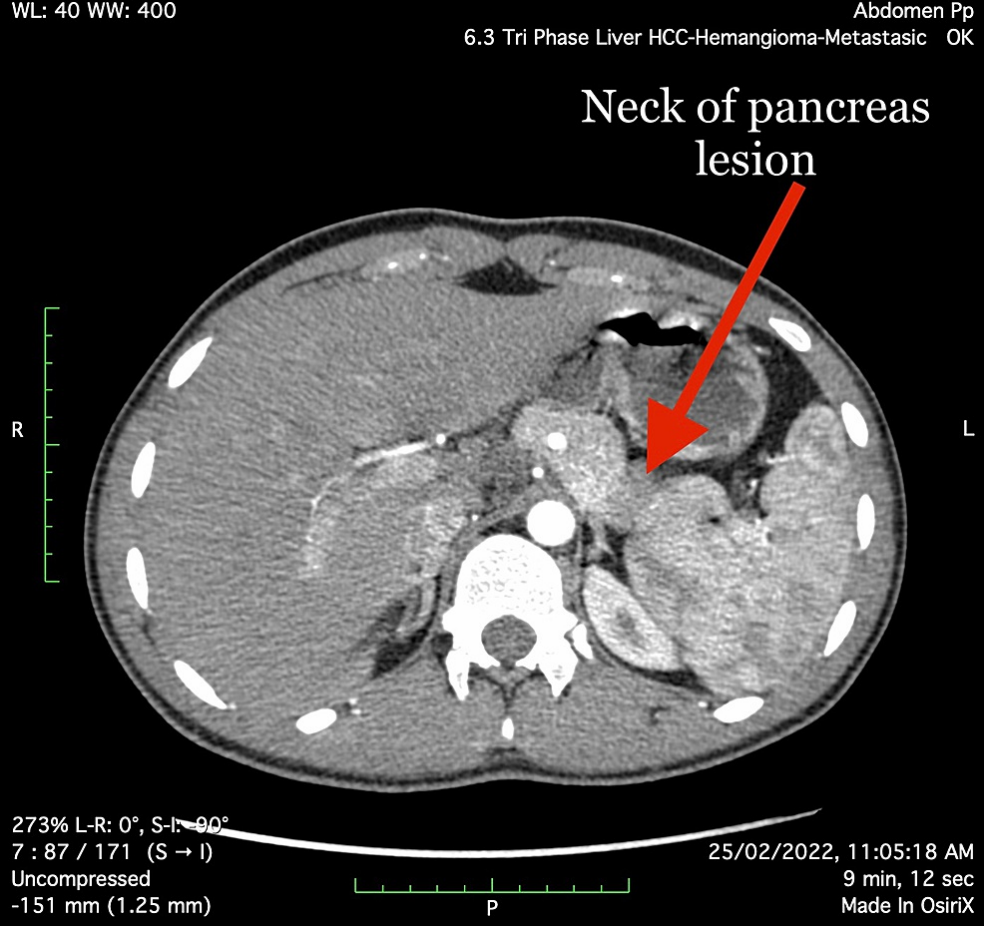
Neck of the pancreas lesion (arterial phase)

**Figure 2:**
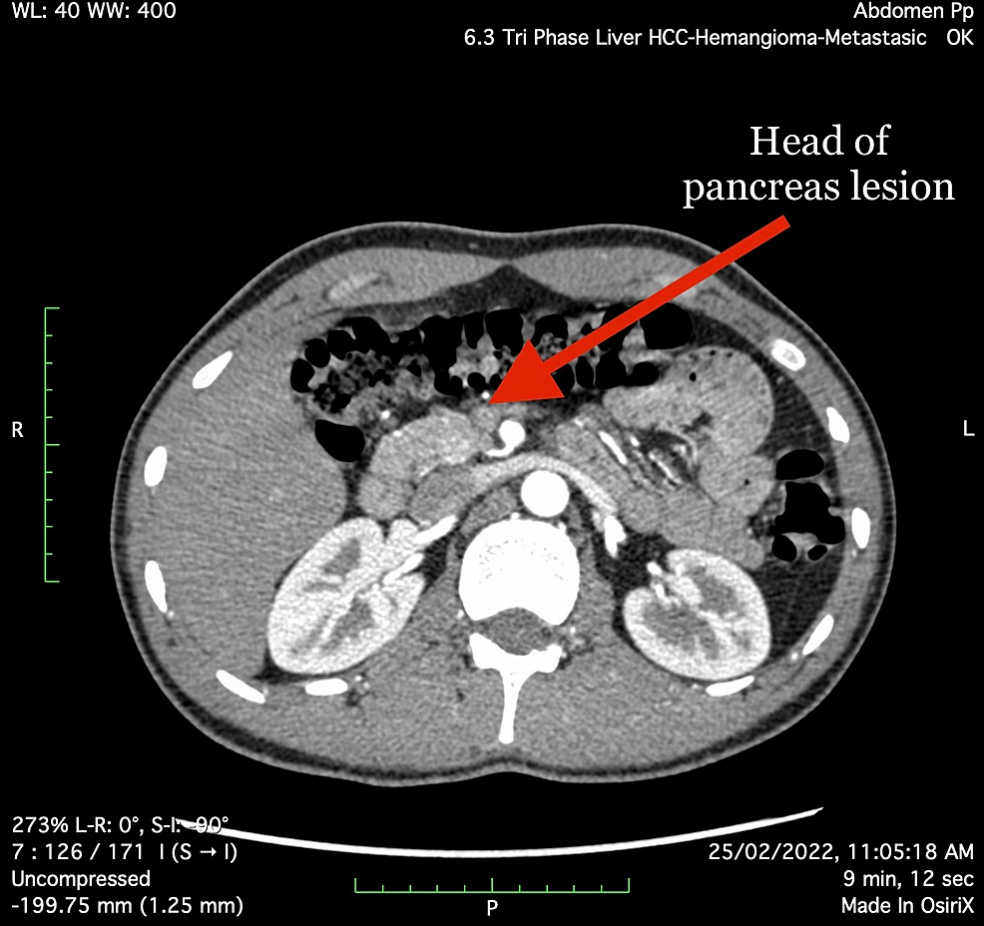
Head of the pancreas lesion (arterial phase)

An abdominal CT scan revealed a rounded arterialized lesion measuring 12x10 mm in the posterior aspect of the pancreas’ neck (Figure 1), as well as an ill-defined hyper-enhancing region measuring 16x14 mm in the pancreas’ head (Figure 2). Given the clinical history, both foci pointed to pancreatic Insulinoma.

The diagnosis of multiple endocrine neoplasia type 1 (MEN1) was excluded based on history, physical examination, and laboratory Investigations (i.e., no history of dyspepsia, or other symptoms of GI ulcers, bone pain or renal stones, tunnel vision, and diplopia were found in this patient). Serum prolactin was 11 ng/mL, PTH was 23 pg/mL, and serum calcium 9.2 was mg/dL. All these normal investigations rule out the diagnosis of MEN1.

The patient was referred from our medical unit to a laparoscopic surgeon for tumor removal via Whipple's procedure.

## Discussion

An insulinoma is a neurosecretory tumor that results in the overproduction of insulin, leading to a state of hypoglycemia. The majority of insulinomas are benign and of solitary character. Due to the "rule of 10," i.e., 10% demonstrate multiplicity, 10% are connected with MEN1, 10% are malignant, and 10% are ectopic [7]. The sign and symptoms of an insulinoma are fasting hypoglycemia, which presents with autonomic symptoms, also termed “sympathoadrenal symptoms,” and with occurrences of neuroglycopenia. In some circumstances, postprandial hypoglycemia is the sole manifestation [8]. In Insulinoma individuals, hypoglycemia is generally explained by decreasing hepatic glucose output rather than increasing glucose use [9]. Insulinomas occur from the ductular/acinar system of the pancreas instead of neoplastic development of islet cells [10]. Pathophysiology by which insulinomas sustain high insulin levels in the condition of hypoglycemia is not known; nevertheless, a study found an improved translation efficiency of a variation of insulin mRNA in large amounts in comparison to ordinary islets of Langerhans [11].

The diagnosis of insulinoma is on the basis of the Whipple triad, which includes symptoms of neuroglycopenia (e.g., behavior change, confusion, irritability) and sympathoadrenal symptoms (e.g., palpitation, tremors) in the existence of low serum glucose level (<50 mg/dL) that resolves following the administration of glucose [12].

The prevalence of insulinoma is reported to be about 1 to 4 per million per year [1,13]. The sign and symptoms of neuroglycopenia include agitation, disorientation, and alterations in vision and behavior, while sympathoadrenal symptoms include palpitations, sweating, and tremulousness [8,12]. Weight gain was described in 18% of patients [14]. Overall, symptoms of hypoglycemia occur only in the fasting state in 735 of patients, whereas 21% reported mixed fasting and postprandial symptoms and 6% documented only postprandial symptoms [8].

A range of techniques is applied to localize an insulinoma, which includes transhepatic portal venous sampling, arteriography, transabdominal ultrasonography, intraoperative ultrasonography, abdominal computed tomography (CT) scan, magnetic resonance imaging (MRI), transhepatic portal venous sampling, endoscopic ultrasonography (EUS), intraoperative palpation, selective arterial calcium stimulation with hepatic venous sampling, and 111 In-labeled octreotide scans with single-photon emission tomography. The sensitivity of some of the methods such as MRI, CT scan, and EUS reported is 37-71%, 30-66%, and 37-71%, respectively, for pancreatic insulinoma identification, whereas the combination of MRI, CT scan, and EUS has reported to identify insulinoma is 17-66%. In addition, arteriography was regarded the gold standard for insulinoma localization. However, documented sensitivities of 29-64%, paired with superior noninvasive imaging techniques, have restricted its use [15].

Overall, the survival of people having insulinoma is high in the long term, and it is estimated that roughly 90-95% of insulinomas have benign histological behavior; therefore, resolution with the absence of symptomatology after full excision is the rule [16]. Among 120 patients in the series of age 4-17 years, only 5.4% cases had detected recurrences [17]. Surgery was carried out in these cases.

In malignant insulinomas, the long-term survival is imaginable, mainly in instances firmly limited to the pancreas and with strong response to adjuvant chemotherapy; median disease-free survival after curative excision is estimated at five years. The median survival time observed after the recurrence of illness is 19 months [16].

## Conclusions

Insulinoma is a sporadic tumor that is more often found in obese patients. Due to increased appetite and repeated episodes of hypoglycemia, patients ordinarily have hyperphagia, leading to obesity. However, our patient with insulinoma is uncharacteristically lean with a BMI of 22. In this way, doctors ought to have a large index of suspicion in patients presenting with repetitive hypoglycemic events that resolve with meals.
